# Internet addiction: Gender-associated differences in psychological characteristics

**DOI:** 10.1192/j.eurpsy.2022.2114

**Published:** 2022-09-01

**Authors:** P. Ponizovsky, E. Skurat, A. Trusova, A. Shmukler, S. Grechany, R. Ilyuk, V. Soldatkin, A. Yakovlev, A. Kibitov

**Affiliations:** 1 Serbsky National Medical Research Center on Psychiatry and Addictions, Department Of Therapy Of Mental Disorders Complicated By Addiction Diseases, Moscow, Russian Federation; 2 Bekhterev National Medical Research Center for Psychiatry and Neurology, Department Of Addictology, Saint Petersburg, Russian Federation; 3 Saint Petersburg State University, Department Of Medical Psychology And Psychophysiology, Saint Petersburg, Russian Federation; 4 Serbsky National Medical Research Center on Psychiatry and Addictions, Department Of Psychiatry, Moscow, Russian Federation; 5 Saint Petersburg State Pediatric Medical University, Department Of Psychiatry And Addictology, Saint Petersburg, Russian Federation; 6 Rostov State Medical University, Department Of Psychiatry, Addictology And Medical Psychology, Rostov-on-Don, Russian Federation; 7 Lipetsk Regional Addiction Hospital, Department Of Addictology, Lipetsk, Russian Federation; 8 Serbsky National Medical Research Center on Psychiatry and Addictions, Molecular Genetics Laboratory, Moscow, Russian Federation

**Keywords:** internet addiction, psychological characteristics, Gender differences

## Abstract

**Introduction:**

Internet addiction (IA) is reported to cause significant negative psychosocial consequences. The gender specificity of psychological characteristics that are potentially significant for the formation of IA remains understudied.

**Objectives:**

To identify gender-related differences in the psychological characteristics of people with IA.

**Methods:**

100 subjects aged 16-34 years who scored 65 points or more on the Chinese Internet Addiction Scale (CIAS) were identified and divided into 2 groups by gender: group 1 (54 men) and group 2 (46 women). The individual psychological characteristics were assessed with: the Liebowitz Social Anxiety Scale (LSAS); the Bass-Perry Aggression Questionnaire (BPAQ); the Emotional Regulation Questionnaire (ERQ); the Adverse Childhood Experiences International Questionnaire (ACE-IQ); a short version of the Five-factor Personality Questionnaire (TIPI-RU) and the Cloninger Temperament and Character Inventory (TCI-125).

**Results:**

Women were significantly more likely to experience fear of situations of interpersonal contact and action in public places (p=0.027). They experienced significantly more sexual violence in childhood (p=0.032) and were more likely to have personality traits such as “reward dependence” (p=0.002), “persistence” (p = 0.046), and “self-transcendence” (p=0.002). Men demonstrated physical aggression (p=0.009), suppressed emotions (p=0.019) significantly more often than women and characterized themselves as emotionally stable (p=0.048).

**Conclusions:**

The gender differences identified in the cohort of individuals with IA can potentially be considered specific for this contingent, although such gender relationships can be observed in other forms of addiction and in the general population. The specificity of gender differences may reflect individual psychological markers of increased vulnerability to developing IA.

**Disclosure:**

The study was financially supported by Russian Foundation for Basic Research within the framework of scientific project No 18-29-22079.

